# Comparative genomics of *Lactobacillus crispatus* suggests novel mechanisms for the competitive exclusion of *Gardnerella vaginalis*

**DOI:** 10.1186/1471-2164-15-1070

**Published:** 2014-12-05

**Authors:** Teija Ojala, Matti Kankainen, Joana Castro, Nuno Cerca, Sanna Edelman, Benita Westerlund-Wikström, Lars Paulin, Liisa Holm, Petri Auvinen

**Affiliations:** Institute of Biotechnology, University of Helsinki, Viikinkaari 4, PO Box 56, FI-00014 Helsinki, Finland; Institute for Molecular Medicine Finland (FIMM), University of Helsinki, Tukholmankatu 8, PO Box 20, FI-00014 Helsinki, Finland; Centre of Biological Engineering (CEB), Laboratory of Research in Biofilms Rosário Oliveira (LIBRO), University of Minho, Campus de Gualtar, 4710-057 Braga, Portugal; Functional Foods Forum, University of Turku, Turku, FI-20520 Finland; Department of Biosciences, Division of General Microbiology, University of Helsinki, Viikinkaari 9c, PO Box 56, FI-00014 Helsinki, Finland; Department of Biosciences, Division of Genetics, University of Helsinki, Viikinkaari 5, PO Box 56, FI-00014 Helsinki, Finland

**Keywords:** Comparative genomics, Lactobacillus crispatus, Pan-genome, Core genome, Normal flora, Bacterial vaginosis, Gardnerella vaginalis, Competitive exclusion

## Abstract

**Background:**

*Lactobacillus crispatus* is a ubiquitous micro-organism encountered in a wide range of host-associated habitats. It can be recovered from the gastrointestinal tract of animals and it is a common constituent of the vaginal microbiota of humans. Moreover, *L. crispatus* can contribute to the urogenital health of the host through competitive exclusion and the production of antimicrobial agents. In order to investigate the genetic diversity of this important urogenital species, we performed a comparative genomic analysis of *L. crispatus*.

**Results:**

Utilizing the completed genome sequence of a strain ST1 and the draft genome sequences of nine other *L. crispatus* isolates, we defined the scale and scope of the pan- and core genomic potential of *L. crispatus*. Our comparative analysis identified 1,224 and 2,705 ortholog groups present in all or only some of the ten strains, respectively. Based on mathematical modeling, sequencing of additional *L. crispatus* isolates would result in the identification of new genes and functions, whereas the conserved core of the ten strains was a good representation of the final *L. crispatus* core genome, estimated to level at about 1,116 ortholog groups. Importantly, the current core was observed to encode bacterial components potentially promoting urogenital health. Using antibody fragments specific for one of the conserved *L. crispatus* adhesins, we demonstrated that the *L. crispatus* core proteins have a potential to reduce the ability of *Gardnerella vaginalis* to adhere to epithelial cells. These findings thereby suggest that *L. crispatus* core proteins could protect the vagina from *G. vaginalis* and bacterial vaginosis*.*

**Conclusions:**

Our pan-genome analysis provides insights into the intraspecific genome variability and the collective molecular mechanisms of the species *L. crispatus*. Using this approach, we described the differences and similarities between the genomes and identified features likely to be important for urogenital health. Notably, the conserved genetic backbone of *L. crispatus* accounted for close to 60% of the ortholog groups of an average *L. crispatus* strain and included factors for the competitive exclusion of *G. vaginalis*, providing an explanation on how this urogenital species could improve vaginal health.

**Electronic supplementary material:**

The online version of this article (doi:10.1186/1471-2164-15-1070) contains supplementary material, which is available to authorized users.

## Background

Lactobacilli are an abundant and heterogeneous group of lactic acid bacteria which occupies a wide variety of carbohydrate-rich niches ranging from plant and dairy environments to host-associated habitats. They reside in the oral cavity, gastrointestinal tract (GIT), and genitourinary tract (GUT) of vertebrates. Lately, their occurrence and activity in the human microbiota as well as their potential biotherapeutic effects have gained substantial interest [1, 2]. The healthy human vagina, for instance, is predominantly colonized by lactobacilli that have a profound impact on the health of women by protecting the host from aberrant urogenital conditions [3–6].

*Lactobacillus crispatus* is an important urogenital species that is routinely found in the vaginas of healthy women [7–9]. It can account for more than 80% of all vaginal bacteria [8] and is considered to be one of the most active species in a healthy vagina [10]. *L. crispatus* also contributes to the maintenance of normal vaginal microbiota, while its absence has been associated with a range of vaginal abnormalities, especially bacterial vaginosis (BV) [10–12]. Strains of *L. crispatus* are even considered as biotherapeutic agents for reducing recurrent urinary tract infections (RUTI) and BV in women [4–6] and have been shown to inhibit *in vitro* the growth, viability, and adhesion of uropathogens [13–16], suggesting a role for *L. crispatus* in protecting the vagina from invading pathogens. Specifically, *L. crispatus* was recently identified to reduce the adhesion of both commensal and pathogenic *Gardnerella vaginalis* to HeLa cells [17], indicating that competitive exclusion of this BV-associated species could be in key role in the health-promoting effects of *L. crispatus.* Besides the GUT, *L. crispatus* has been detected in the GIT of animals. The species is among the most profuse lactobacilli in the chicken crop [18] and has, for example, been isolated from the stratified squamous epithelium of the non-secreting portion of the horse stomach [19] and the feces of pigs [20]. *L. crispatus* has also been recovered from human fecal samples [21, 22], but this result is best explained by its presence in oral cavity and rectum [23, 24]. Intriguingly, the rectal reservoirs of *L. crispatus* have been associated with a lower prevalence of BV [24, 25], suggesting the role of rectal *L. crispatus* in the maintenance of the healthy vaginal flora [25].

Recently, the genome sequences of ten *L. crispatus* strains have become publicly available [26, 27]. The genomes are all about 2.0–2.7 Mb in size, with a GC content of ~37%. They possess a large number of tRNA molecules (45 to 64) and are predicted to encode 2,022–2,643 proteins, several of which are of potential importance to vaginal health. For example, the potential to inhibit harmful microorganisms by direct inhibition through lactic acid, hydrogen peroxide, and bacteriocins or by displacing them through competitive adhesion is supported by the genome annotation data. In addition, these genomes have verified the phylogenetic position of the species in the *Lactobacillus delbrueckii* clade [28, 29]. Out of the ten *L. crispatus* strains having had their genome defined, nine are vaginal isolates and were sequenced as a part of the Human Microbiome Project [26], including the strain CTV-05 that may have a role in the treatment and prevention of BV and RUTI [4–6]. The remaining genome belongs to the chicken-isolated strain ST1 [27], known for its strong adherence not only to chicken epithelia but also to buccal and vaginal cells of human origin [30–32]. The strain ST1 was recently also characterized to produce a *Lactobacillus* epithelium adhesin (LEA) that displays specific binding to both crop epithelium and epithelial cells from human vagina [33].

Thus far, the genome sequences of different *L. crispatus* strains have been studied separately. Unfortunately, a single genome sequence may not reflect the entire genomic complement of a species or provide an understanding of the biological processes that are peculiar to the species. Instead, better knowledge of the genetic diversity of a bacterial species can be gained by comparative genomics [34–40]. For example, comparative genomic analyses have established considerable intraspecies genetic diversity within the *L. delbrueckii* clade [34–37], but have also unraveled specific mechanisms of the host-microbe interaction that are common for all strains of the given species [38–40], suggesting species-specific rather than strain-specific host interaction properties. In the present study, we used comparative genomics to assess the overall genomic similarity of ten *L. crispatus* strains and defined their core and pan-genome. This global view on the gene content of *L. crispatus* provided an accurate account of features associated with vaginal health and represents the first effort to describe the genomic potential of this central urogenital species. Specific focus was placed on the molecular mechanisms governing host-microbe and microbe-microbe interactions. These mechanisms involve genes encoding or implicated in the production of antimicrobial peptides, adhesion-associated compounds, exopolysaccharide (EPS), and S-layer proteins forming a paracrystalline structure on the cell surface [41–43]. In addition, *L. crispatus* ortholog data was compared and contrasted with that of *G. vaginalis,* a frequent and predominant colonizer of the vagina of women with BV [44, 45], implicated also in the development of the disease [46]. These analyses revealed collective molecular factors in *L. crispatus* antagonistic to *G. vaginalis*, such as a counterpart to a *G. vaginalis* major subunit pilin*.* The detected factors provided an explanation for the previously reported ability of *L. crispatus* to reduce the adhesion of *G. vaginalis* to host cells [17] and for the inverse association between *L. crispatus* and *G. vaginalis* colonization in the vagina [12, 44, 47]. Overall, this pan-genome study of *L. crispatus* broadens our knowledge of this central vaginal colonist and sheds light on the molecular mechanisms by which *L. crispatus* could prevent BV and protect the vagina from pathogens.

## Materials and methods

### Genome entries and strains

All available genome sequences of *L. crispatus* in public databases as of January 2013 were included in this work (Table 1). In addition, all available genome sequences of *G. vaginalis* with annotated coding sequences (CDSs) in their genome files as of May 2014 were included in the *G. vaginalis* genome analyses (Additional file 1). To resolve the phylogenetic position of *L. crispatus* in respective to closely related lactobacilli, genomes of *Lactobacillus helveticus, Lactobacillus acidophilus* and *Bacillus subtilis* were downloaded and analyzed together with the *L. crispatus* genomes. The set of *L. helveticus, L. acidophilus* and *B. subtilis* genomes included in this phylogenetic analysis is listed in Additional file 2. The annotated genomes were retrieved in GenBank format from GenBank [48] or PATRIC [49]. For the draft genomes, supercontigs were preferred, if available.

**Table 1:** **Overview of** ***L. crispatus*** **strains, properties and main findings**

Strain & accession	Scaffolds	Plasmid derived contigs	Genome size (Mb)	Conserved genome (%)	CDS	Ortholog groups	Average CDS length	CRISPR/Cas system	Prophage clusters	GIs	Adhesins	Comments
125-2-CHN [GenBank:ACPV00000000]	30	0	2.31	86	2082	2050	902	Type II	1	6	9	Vaginal isolate from a healthy Chinese woman
214-1 [GenBank:ADGR00000000]	187	1	2.07	97	2163	2135	847	Type II	2	7	10	Human vaginal isolate
CTV-05 [GenBank:ADML00000000]	25	0	2.36	90	2248	2032	838	Type II	6	8	9	Human vaginal isolate; strong adhesion to vaginal cells; health promoting use in treatment and prevention of BV and RUTI
FB049-03 [GenBank:AGZF00000000]	5	0	2.46	92	2433	2332	848	Type II	3	8	11	Human vaginal isolate
FB077-07 [GenBank:AGZG00000000]	10	1	2.70	84	2643	2516	837	Type II	3	21	13	Human vaginal isolate
JV-V01 [GenBank:ACKR00000000]	86	0	2.22	90	2209	2180	839	Type II	2	5	12	Human vaginal isolate
MV-1A-US [GenBank:ACOG0000000]	45	0	2.31	90	2151	2126	877	Type II	6	8	9	Vaginal isolate from a healthy US woman
MV-3A-US [GenBank:ACQC00000000]	76	0	2.44	89	2330	2275	859	Type II	6	14	9	Vaginal isolate from a healthy US woman
SJ-3C-US* [PATRIC:ADDT00000000]	201	1	2.09	97	2174	2138	821	Type II	1	15	10	Vaginal isolate from a healthy US woman
ST1 [GenBank:NC_014106]	1	0	2.04	82	2022	1957	896	Type I	0	10	11	Chicken isolate; adheres also to human vaginal cells; inhibition of APEC adhesion

*Annotations are derived from PATRIC.

BV: Bacterial vaginosis; RUTI: Recurrent urinary tract infection; APEC: Avian pathogenic *Escherichia coli.*

For adhesion assays, *G. vaginalis* strain 101 isolated from a woman with BV [50] and a vaginal *Lactobacillus crispatus* strain EX533959VC06 isolated in the scope of the project “The Vaginal Microbiome: Disease, Genetics and the Environment” of the Human Microbiome Project [26] were used.

### Reference-based genome scaffolding

The draft genomes of *L. crispatus* (strains 125-2-CHN, 214-1, CTV-05, FB049-03, FB077-07, JV-V01, MV-1A-US, MV-3A-US, and SJ-3C-US)*, L. helveticus* (strains DSM 20075 and MTCC 5463), and *L. acidophilus* (strain ATCC 4796) were subjected to reference-based genome scaffolding using progressive Mauve genome alignment software with default settings [51]. The genome sequences of the strains ST1, DPC 4571, and NCFM served as references for the *L. crispatus, L. helveticus*, and *L. acidophilus* draft genomes, respectively. The contig order was confirmed through whole genome sequence comparisons that were generated using BLASTN [52], and visualized using the Artemis Comparison Tool (ACT) [53]. Putative plasmid-derived contigs among *L. crispatus* genomes were separated from chromosome derived sequence fragments using cBar with default settings [54]. Potential plasmid-derived contigs 2.5 kb or longer were then extracted and aligned to known plasmid sequences using PATRIC’s BLASTN [49]. Contigs that aligned at ≥40% identity over ≥70% of their length were considered as plasmid-derived.

### Phylogenetic analyses

The organized scaffolds of the 18 strains of *L. crispatus, L. helveticus,* and *L. acidophilus* were aligned using Mauve Progressive Aligner [51]. Fully conserved columns with single nucleotide polymorphism (SNP) were extracted with Mauve genome alignment software [51], and used for the construction of the phylogenetic tree using PhyML with default settings [55]. Maximum-likelihood trees were visualized with iTOL [56]. For correct rooting of the phylogenetic tree, a SNP-based phylogenetic tree including the *B. subtilis* genome as an out-group was constructed using the same approach.

### Genome re-annotation

In order to ensure the identical quality standards for all the investigated genomes, a functional annotation update was performed for *L. crispatus* CDSs. Additional annotation information for the CDSs was obtained with Blannotator [57], best BLAST, Rast [58], the automatic annotation server (KAAS) [59], COG functional classification system [60], and by searching the predicted protein products against the PFAM database release 26.0 [61]. For Blannotator and best BLAST approach, BLASTP was run with default parameter settings, and hits that aligned with more than 40% amino-acid identity and 80% coverage threshold were retained. The Rast [56] and KAAS [57] and COG [58] annotation was obtained using the services with default settings. PFAM searches were performed locally using the HMMer 3.0 package [62], relying on the PFAM trusted cut-off for the score. The EPS gene clusters were identified by manual examination of the annotation information. The presence of putative bacteriocin-encoding genes was determined with BAGEL3 [63] with default settings. To identify genes associated with clustered regularly interspaced short palindromic repeats (CRISPRs), CDSs were screened for the presence of CRISPR-associated (Cas) protein domains using the hmmscan program from the HMMer 3.0 package [62]. Matches having scores exceeding the trusted cut-off values were considered significant. Cas protein domain models were obtained from the TIGRFAM database [64, 65]. Integration of annotation information was done using *in*-house perl scripts producing tab-delimited CDSs information files.

Other bioinformatic analyses included identification of mobile genetic elements and CRISPR loci. Genomic regions potentially obtained by horizontal gene transfer (HGT) were predicted using IslandPick, IslandPath-DIMOB and SIGI-HMM methods with the help of IslandViewer meta-analysis tool with default settings [66]. Prophage-like gene-clusters were predicted with Prohinder using default parameters [67]. Overlapping prophage-like genome regions were merged into single extended regions spanning a given genomic region and manually inspected. Putative CRISPR loci were identified with PilerCR run with default settings [68] and manually adjusted. MegaBLAST (default parameters) [52] was used for similarity searches between CRISPR-spacer sequences and virus (taxid:10239) and plasmid (taxid:36549) entries in the GenBank database. Only matches showing 100% identity over the complete CRISPR-spacer were retained.

### Annotation of proteinaceous adhesion factors

*L. crispatus* CDSs potentially involved in binding to the host were identified by searching the predicted protein sequences against adhesion associated PFAMs. Adhesion associated PFAMs were identified by searching the PFAM database release 26.0 [61] entries with various keywords related to adhesion, host tissue components, and bacterial surface components, and by manual examination of the literature. The list of PFAM domains is available in Additional file 3. In addition, non-adhesion related domains for the selected adhesion-related CDSs were detected by searching the protein sequences against PFAM release 27.0 through the PFAM website using gathering thresholds greater than or equal to the trusted cut-off.

### Ortholog prediction

Ortholog groups among *L. crispatus* strains were identified using OrthoMCL [69]. To estimate the development of the size of the core and pan-genome as a function of the number of sequenced *L. crispatus* strains, ortholog groups were determined iteratively for an increasing numbers of sequenced genomes. At each sample size, the analysis was repeated 50 times with different random sets of *L. crispatus* genomes. OrthoMCL was run with default settings, except for a percent match threshold of 35 and BLASTP set to print up to 10,000 alignments. The protein products of the original CDSs were used for the analysis. The same approach, but without the sampling procedure, was used to define the ortholog groups among *G. vaginalis*. Because of the draft quality of most of the *G. vaginalis* genomes, ortholog groups present in ≥ 30 *G. vaginalis* genomes were considered as core groups.

### Estimation of *L. crispatus*pan- and core genome sizes

The estimation of the *L. crispatus* core and pan-genome sizes was based on the OrthoMCL results and was performed according to previously described approaches [70]. The core genome was extrapolated by fitting an exponential decaying function *y* = *κ* exp(-*N*/*τ*) + *Ω* to the median number of core ortholog groups with a weighted least square regression. In the equation, *N* is the number of sequenced strains and *κ*, *τ,* and *Ω* are free parameters optimized in the regression analysis. The *Ω* describes the estimated core genome size. The power law *y* = *k N*^*β*^ was fitted to pan-genome data with a weighted least square regression, where *y* is the median, *N* is the number of genomes, and *k* and *β* are free parameters. Regression analyses were done using the *nls* function of the statistical software R [71].

### Identification of significant enrichment of genes in COG-categories

Hypergeometric distribution was used to test the probability of the over-representation of core, strain-specific or variably conserved accessory genes in a given cluster of orthologous groups (COG). The obtained *p*-values were subjected to Bonferroni adjustment to reduce the number of false positives introduced by multiple hypothesis testing. Only COG categories containing more than 20 CDSs were included in the analysis. Statistical tests were performed using the statistical software R [71].

### Identification of antagonistic factors against *G. vaginalis*

Virulence-related *G. vaginalis* CDSs were inferred from a recent comparative genomic analysis [72] and by comparison to the PFAM database [61]. The PFAM search was done using the hmmersearch program from the HMMer 3.0 package. Hits were considered significant if their score was above a trusted cut-off value. Virulence-related PFAM models were identified based on a literature review. Following the identification of *G. vaginalis* virulence factors, all the members of their ortholog groups were extracted, an alignment built using Muscle with default settings [73], and a hidden Markov model (HMM) constructed using the hmmbuild command. The constructed HMMs were then searched against the predicted *L. crispatus* proteomes with the hmmersearch program from the HMMer 3.0 package in order to identify counterparts. Hits with E-value greater than or equal to 0.01 were accepted and manually inspected.

### Detection of enzymes and metabolic pathway reconstructions

Using the automatic annotation server KAAS [59], *L. crispatus* CDSs were assigned with EC numbers describing enzymatic activity. Each strain’s ability to ferment carbohydrates and synthetize bio-compounds was then tested by matching its EC complement against the sets of ECs of metabolic reactions providing the conversion of a given starting compound to a particular end product. A route was accepted as intact if at least one match was found for each enzyme-catalyzed reaction. Metabolic routes between two given compounds were retrieved from the FMM server [74] which connects different KEGG reference reaction maps [75] and reconstructs metabolic pathways between metabolites. For the analysis, the amino acids were paired with amino acid synthesis starting materials and with each other; carbohydrates were paired with selected key intermediates of the central carbon metabolism; the selected central carbon metabolism intermediates were paired with pyruvate or pyruvate, acetate and ethanol; and pyruvate was paired with various end products. The exact list of compound pairs screened is available in Additional file 4. To determine pathways encoded by the *L. crispatus* core genome, the above pathway reconstruction approach was repeated for the core genome-encoded EC complement. Finally, the mode of carbohydrate fermentation was studied based on MetaCyc pathways for homolactic and heterolactic fermentation [76]. Hydrogen peroxide generating enzymes were detected by screening for EC numbers of the enzymes having the compound H_2_O_2_ (C00027) as a product.

### Adhesion assays

Bacteria were grown in supplemented brain heart infusion (Oxoid) containing 2% (w/w) gelatin (Oxoid), 0.5% yeast extract (Liofilchem), 0.1% starch (Fisher Scientific) and 0.1% glucose (Liofilchem), for 48 h at 37°C, in 10% CO_2_. Bacterial suspensions were collected by centrifugation at 6,960 g at 4°C for 10 min and washed once with sterile phosphate buffered saline (PBS). Bacteria were resuspended in PBS and the optical density at 600 nm (OD600) was determined. Correlations between OD600 and Colony Forming Units (CFUs) were made prior to the experiments, and the bacterial suspensions were adjusted to 1 × 10^8^ CFUs/mL, as optimized before [17].

For the adhesion assays, HeLa cells (American Tissue Culture Collection, ATCC CCL-2) were cultured in DMEM supplemented with 10% (vol/vol) fetal bovine serum (Sigma-Aldrich) and 1 IU penicillin-streptomycin/mL (Sigma-Aldrich) at 37°C and in 5% CO_2_. Cells were cultured in chamber slides (Lab-Tek) until they reached a density of 2 × 10^5^ cells per well (≈ 90% confluence), at 37°C in 5% CO_2_. Before the adhesion assays, cells were washed twice with 200 μL of PBS to remove non-adherent cells and fixed with cold 4% (w/v) paraformaldehyde (PFA; Santa Cruz Biotechnology, Inc.) in PBS for 10 min followed by washing three times with PBS.

Fab fragments prepared by papain treatment of purified IgG against LEA protein of *L. crispatus* ST1 and flagellum of *Escherichia coli* strain MG1655 Δ*fimA-H* were available from a previous study [33]. Fab fragments (final concentration 0.7 mg/mL) in PBS supplemented with 5 mM phenylmethylsulfonyl fluoride (PMSF; Sigma-Aldrich) were mixed in independent experiments with *G. vaginalis* or *L. crispatus* cells, at room temperature, for 30 min, with rotational agitation at 0.028 g. Mixtures of Fab fragments and bacteria or bacteria alone in PBS supplemented with 5 mM PMSF were incubated with PFA-fixed HeLa cells for 1 hour, at 37°C in 5% CO_2_. Each well was carefully washed twice with 200 μL of sterile PBS to remove non-adherent bacteria. Bacterial quantification was done as previously described [77]. Briefly, after fixing with methanol, DAPI (2.5 μg/mL; Sigma-Aldrich) was added to the wells. Microscopic visualization was performed using an Olympus BX51 epifluorescence microscope equipped with a CCD camera (DP72; Olympus) and filters capable of detecting the DAPI staining (BP 365–370, FT 400, LP 421). The number of adherent bacteria in 20 randomly chosen microscope fields was determined using *Image J* software (version 1.41). Results were expressed as the bacteria per HeLa cells, according the mean ± standard deviation of the two independent experiments, with technical duplicates. The data were analyzed using the Student’s *t*-test with the statistical software package SPSS 17.0 (SPSS Inc. Chicago, IL). *P*-values of less than 0.05 were considered significant.

## Results and discussion

### General genomic features of *L. crispatus*

**Figure 1:**
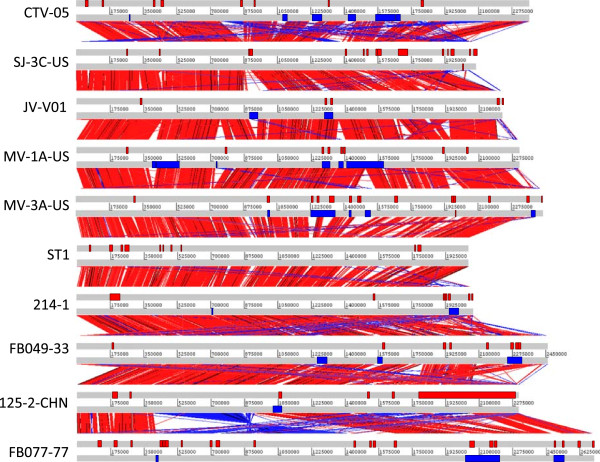
**Whole-genome alignment of the** ***L. crispatus*** **genomes.** The contigs of the draft genomes were ordered with MAUVE using the ST1 genome as a reference. Matching genome regions were identified with BLASTN and visualized using the Artemis Comparison Tool (ACT). Vertical bands represent the BLASTN matches (bit score ≥ 1500). Prophinder-predicted prophage-like genomic regions and IslandViewer predicted GIs are represented as blue boxes on the bottom and red boxes on the top strand of each genome, respectively.

The genome sequences of ten *L. crispatus* strains were compared and analyzed (Table 1). These genomes contain 22,455 CDSs, of which 13,774 (61.3%) had an assigned role in the original genome file. After the annotation update, 19,414 CDSs (86.5%) were functionally classified by at least one of the functional annotation tools. For each CDS, the results of the different protein classification analyses were collected together and analyzed as a group. The obtained annotations are presented in Additional file 5. Only one of the genomes (ST1) is in one contig whereas the rest are in 5–201 super-contigs. Putative plasmid-derived sequences, each with a length of 2,000 bases or more were identified in three vaginal isolates (214-1, FB077-07 and SJ-3C-US), the rest having only chromosomal-associated super-contigs. Using conserved genomic synteny, the orientation and order of the chromosomal-associated super-contigs of each draft genome was determined. Analyses of the resulting architecture revealed that genomes were in general collinear (Figure 1) and shared on average ~90% of each other’s content, comparable to conservation ratios seen in *Lactobacillus johnsonii* [35], *L. helveticus* [34], and *L. plantarum* [78]. The genomes of the strains 214-1 and SJ-3C-US were most conserved, with ~97% of their sequences conserved in at least one strain, whereas only roughly 82% and 84% of the genomes of strains ST1 and FB077-07 could be aligned against some other *L. crispatus* genome (Table 1). These data indicate that each assembly presents a near complete chromosome, providing a solid foundation for inter-strain comparisons.

### The *L. crispatus*pan-genome

**Figure 2:**
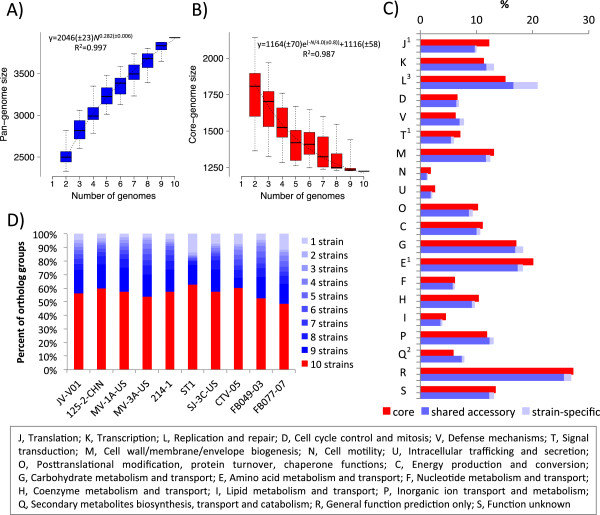
**Pan- and core genomes of** ***L. crispatus*** **.** Development of the pan- **(A)** and core **(B)** genomes as a function of the number of sequenced *L. crispatus* strains. The total number of genes found according to the pan- and core genome analysis is shown for increasing numbers of sequenced genomes. The dashed lines represent least squares fits to the medians and the *R* ^*2*^ describes the suitability of the fit. The box plots present median (horizontal line), 25th and 75th percentiles (solid box), with the data extremes shown by whiskers outside the box. **C)** The distribution of core and accessory *L. crispatus* CDSs within COG functional categories. For each category, the top and bottom bars show the percentage of the assigned core and accessory CDSs relative to the entire core and the accessory *L. crispatus* CDSs, respectively. The proportion of the strain-specific CDSs is highlighted (light blue) in the accessory bars. COGs significantly enriched (*p*-value ≤ 0.01, hypergeometric distribution) in core (1), shared accessory (2), or strain-specific (3) CDSs are marked next to the COG identifiers. Only COG functional categories with more than 20 members are shown. The COG categories are given in the inset at the bottom of the figure. **D)** Distribution of ortholog groups at different levels of conservation in each strain. The OrthoMCL-defined ortholog groups were classified into different levels of conservation according to the number of strains they were detected in. Ortholog groups found in all the ten genomes represent the current core (red). Conservation levels are represented by different colors.

The microbial pan-genome is defined as the full complement of genes in a species [79]. In total, this set of *L. crispatus* genomes comprised 3,929 ortholog groups, including on average 5.2 orthologs and 0.2 co-orthologs per group. This current pan-genome was defined using OrthoMCL and was almost twice the average number of CDSs (~2,250) and ortholog groups (~2,170) present in a single *L. crispatus* strain (Table 1). The ortholog group accumulation curve describing the expansion of the pan-genome as a function of genomes added to the analysis fitted well a power law model and was far from saturated (Figure 2A), indicating that the total gene pool accessible to the species has not yet been fully captured [79] and suggesting yet-to-be discovered traits in *L. crispatus*, similar to that what has previously been reported for *Oenococcus oeni* [80] or *Lactobacillus paracasei* [38]. Particularly, the regression model [70] revealed an open pan-genome (positive exponent β = 0.282 ± 0.006) that grows by at least ten ortholog groups per every additional genome until 285 isolates have had their genome defined.

### The *L. crispatus*core genome

The core genome is defined as the orthologous genes present in every strain of a species [79]. We identified the current *L. crispatus* core genome to be comprised of 1,224 ortholog groups that were conserved across all the ten analyzed strains. This common core captured ~57% of the ortholog groups of a given genome, which is slightly less than what orthologous grouping has revealed for another *Lactobacillus* species [38]. Based on the examination of the COG functional categories and hypergeometric tests (*p*-value ≤ 0.01), the core was identified to be significantly enriched with genes belonging to COG categories J (translation), T (signal transduction), and E (amino acid metabolism and transport) (Figure 2C). Furthermore, ~10% of the ortholog groups in the core genome could not be assigned with a descriptive functional annotation (Additional file 5), and thus may represent proteins with yet-to-be discovered housekeeping functions or other functions relevant to the basic aspects of the biology of the species. We also predicted the core genome to contain genes encoding features likely to contribute to cell envelope biogenesis, antimicrobial activity, and host-microbe interaction, as illustrated in detail below.

To estimate the number of ortholog groups present in an infinite number of *L. crispatus* strains, the number of shared ortholog groups found on sequential addition of each new genome sequence was extrapolated by fitting an exponential decaying function to the medians of core genome sizes [70]. As expected, the number of ortholog groups in the core genome initially decreased with the addition of each new genome sequence. The extrapolation of the curve designated that the core genome plateaus at 1116 ± 58 ortholog groups for an infinite number of *L. crispatus* strains (Figure 2B). Thus, the current *L. crispatus* core genome appears to be almost within the estimated error margin, indicating that the current core is nearly a perfect representation of the final core genome. However, it should be noted that gaps and sequencing errors in draft assemblies might have affected our estimate [81].

### The *L. crispatus*variome

We investigated the distribution of the *L. crispatus* pan-genome by assessing the number of strains sharing a particular ortholog group (Figure 2D). In total, 2,705 ortholog groups were present in some, but not in all the ten *L. crispatus* strains, forming the current *L. crispatus* accessory genome, suggested to provide selective advantages for different strain(s) of a species [70, 79]. The overall composition of the COGs in the core and accessory genomes was mainly similar (Figure 2C), the most notable (*p*-value ≤ 0.01) over-representations of accessory genome-encoded genes being associated with COG categories L (replication and repair) and Q (secondary metabolites biosynthesis, transport and catabolism). Enrichment in the L and Q categories was driven by diversity in strain-specific transposon-associated classes and ABC-type multidrug transporters, respectively. Included in the accessory genome were also 1,311 ortholog groups found only in a single strain. Most of these ortholog groups belonged to the genomes of the strains FB077-77 and ST1 (287 and 264, respectively), which also displayed the smallest (733) and largest (1,292) accessory gene pools, respectively. Fewest strain-specific groups were present in the genome of the strain MV-1A-US. The mean number of the strain-specific ortholog groups found in the *L. crispatus* dataset was 131 ± 84, which forms a slightly bigger portion of the genome than what comparative analyses have previously detected in another *Lactobacillus* species [82] and less than in some other lactic acid bacteria such as *O. oeni* [80]. As expected, the strain-specific gene pool is poorly characterized, close to 40% lacking a functional annotation. Interestingly, transposase-related genes accounted for ~25% of all strain-specific genes with an informative functional annotation. Protein homology searches revealed that ~30% of all strain-specific genes had the highest similarity to genes found in other strains of the *L. delbrueckii* clade (Additional file 6). The species *L. helveticus* and *Lactobacillus kefiranofaciens* were deduced to be the two most notable reservoirs of genetic variability, providing the best matching targets for about 10% and 5% (respectively) of the strain-specific ortholog groups in *L. crispatus*. For example, up to 47% of strain-specific ortholog groups in strain SJ-3C-US had the top match in *L. helveticus*. In addition, more distant *Lactobacillus* species appear to have interacted with *L. crispatus*. Specifically, the strain ST1 seems to have received seven strain-specific ortholog groups from *Lactobacillus salivarius*, which is only distantly related to *L. crispatus* ST1 but known to exist in the same ecological niche [18].

### Horizontal gene transfer

HGT is a major force in bacterial evolution and can contribute to the fitness, metabolic versatility, and niche-adaptation of bacteria [83]. For example, genomic islands (GI) harboring genes for carbohydrate utilization reflect to the lifestyle adaptation of *Lactobacillus plantarum* [78]. To determine the presence of GIs and potentially horizontally acquired genes, the *L. crispatus* genomes were interrogated using IslandViewer [66]. This analysis identified between 5 and 21 GIs in each genome (Table 1). Some of these GIs agreed with the observed interruptions in the genomic synteny whereas others were conserved (Figure 1), highlighting the imprecision of the prediction methods or indicating the presence of ancient GI acquisition events in *L. crispatus*. The total span of GIs was longest in *L. crispatus* 125-2-CHN (~574 kb), shortest in the strain JV-V01 (~47 kb), and on average ~166 kb in a *L. crispatus* genome. Based on COG and prophage-cluster analysis, over 500 of the total of 1,571 CDSs in the GIs encoded phage-related products or transposases, which is not surprising, given that many of the prophage-like genomic regions co-localize with the GIs (Figure 1). In addition to the mobile elements, the GIs were found to be rich in metabolism and biosynthesis-related genes. Close to 20% of their gene content was predicted to be involved in sugar metabolism and amino acid biosynthesis, pointing a role for HGT in adaptation of *L. crispatus* to varying environments. For example, HGT events may have contributed to acquisition of cellobiose and fructose-specific transport systems as well as genes implicated in sialic acid utilization to certain *L. crispatus* strains (Additional file 5). On the other hand, the more ancient gene acquisition events in *L. crispatus* provide an explanation for the observed presence of an additional copy of phosphoketolase genes missing in the closely related *L. acidophilus* and *L. helveticus* genomes included in the phylogenetic analysis. Similarly, the investigated *L. acidophilus* and *L. helveticus* strains also lacked a GI-associated mannosylglycerate hydrolase encoding genes present in some *L. crispatus* strains. Moreover, missing from *L. acidophilus* genomes were also a hydrogen peroxide producing glycolate oxidase (EC:1.1.3.15) gene that was present in all the *L. crispatus* and most *L. helveticus* genomes, further supporting the role of HGT in environmental adaptation. Another hydrogen peroxide producing enzyme, puryvate oxidase, was in contrast predicted to be present in all except three *L. crispatus*, *L. helveticus*, and *L. acidophilus* genomes. The *L. crispatus* GIs comprised also several putative EPS biosynthesis genes in strains ST1, 125-2-CHN, and FB049-03, which is in accordance with the observation that EPS gene clusters in lactobacilli often have abnormal GC content [84]. Finally, 145 strain-specific genes were associated with GIs. Most of these were distributed somewhat randomly, but it was also possible to define eight long (minimum of five genes) GIs with considerably many strain-specific genes and probably thus acquired rather recently by HGT. In three of these GIs (*EKB62214.1-EKB62134.1, EKB62035.1*-*EKB62043.1* and *LCRIS_01745*-*LCRIS_01757*), the majority of the CDSs did not show significant similarities to proteins in the NCBI databases, suggesting a recent acquisition of yet-undiscovered traits.

### Phages

Temperate phages are common in vaginal lactobacilli and can form a potential threat for *Lactobacillus* populations maintaining a healthy vagina [85–87]. Some studies have even suggested that bacteriophage attack is the causative agent triggering the breakdown of the protective vaginal microbiota during BV [86, 87]. In this study, a total of 31 prophage-like regions were identified comprising of 1,636 CDSs and accounting for more than a fifth of the ortholog groups in *L. crispatus*. Markedly, this fraction of prophage-like ortholog groups in *L. crispatus* is substantially higher than the 9% reported for *L. paracasei* [38], indicating a large variation of prophage-related gene contents among different *Lactobacillus* species. Interestingly, the prophage-like clusters were enriched in the nine vaginal isolates of *L. crispatus*, whereas there was none in the chicken isolate ST1 (Table 1, Figure 1), possibly reflecting exposure to phage in the human vagina. Specifically, the strains 125-2-CHN, SJ-3C-US, 214-1, JV-V01, FB077-07, and FB049-03 each contained between one and three prophage-like regions composed mostly of CDSs with phage-related or non-informative annotations and with no or limited homology with the genome sequence of other *L. crispatus* strains. The remaining three vaginal isolates (MV-3A-US, CTV-05, and MV-1A-US) carried six candidate prophages, each consisting mostly of orphan CDSs with phage-like or non-informative annotations. Sequence analysis of *L. crispatus* ST1 genome also revealed a prophage-like region, but this region was rejected, because associated with the strain’s own replication machinery. Overall, the results are in accordance with the high degree of lysogeny, namely 77%, observed for vaginal *L. crispatus* strains [85]. This suggests that temperate phages are widespread in vaginal lactobacilli and that transduction is an important mechanism for genome evolution in these bacteria. Notably, the lack of common insertion sites between the isolates indicates that various sites of the *L. crispatus* genomes can serve as targets for phage integration (Figure 1).

### CRISPR/Cas-systems

**Figure 3:**
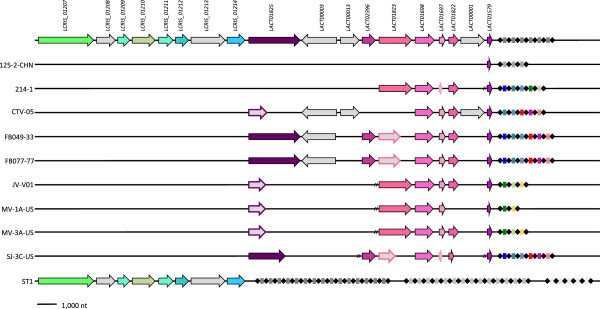
**Variation in CRISPR/Cas locus in** ***L. crispatus.*** The arrows represent different genes and their orientation within a locus. Orthologous genes are positioned vertically. The *cas* genes with conserved function are of the same color, whereas grey describes genes not matching a Cas model. Outlines of genes orthologous to some *cas* gene, but not matching a Cas model are color coded according to the ortholog groups. Dashed lines represent contig breaks. Diamonds represent direct repeats and boxes different spacer sequences. Identical spacers are represented by the same color. The direct repeats of the top nine genomes are identical.

CRISPRs are a family of DNA repeats present in the genomes of many prokaryotes that are responsible for providing acquired immunity to exogenous DNA from bacteriophages and plasmids. This system consists of a set of *cas* genes and an array of direct repeats separated by intervening sequence spacers derived from the invading DNA [64, 88–90]. Interestingly, distinct types of CRISPR/Cas systems were identified for the vaginal *L. crispatus* isolates and the chicken isolated ST1 (Table 2). All the vaginal isolates but the strain 125-2-CHN were predicted to have several genes that could be classified to belong to the previously described Type II CRISPR/Cas system [64]. Analysis of the genome of 125-2-CHN also revealed traces of the Type II system, but the presence of universal *cas1* and *cas2* core genes and the *cas9*/*csn1* signature genes could not be verified, because the region next to *csn2* is disrupted by a sequencing gap. Nevertheless, the CRISPR arrays in each of the vaginal strains was composed of direct repeats with an identical consensus sequence of 36 bp and two to six spacer sequences each. Homology searches between the identified spacers and public virus and plasmid sequences did not reveal the putative targets of these systems, which is in line with the previous spacer annotation survey [90] identifying a plasmid or virus target only for 30% of the spacer sequences in lactic acid bacteria. The lack of identified targets of the *L. crispatus* spacers points to a pool of not yet sequenced vaginal phages and plasmids. Interestingly, many spacers were identical across several of the vaginal strains (Figure 3), suggesting that these strains may share a recent ancestor or have encountered similar invading genetic elements in their past. It should be noted that these genome sequences are incomplete and that some spacers and repeats may have remained undetected. In addition, a Type I CRISPR/Cas system [64] was identified in the ST1 genome comprising eight *cas* genes and three CRISPR-arrays composed of direct repeats of 28 bp, and 14, 15, and 5 spacers. The repeats were highly similar and resembled a repeat discovered in 31 vaginal samples by Rho *et al*. [91]. However, the shortest array was positioned within a 423 bp long putative *LCRIS_01228* gene and thus is most likely a false prediction. Similarly to the vaginal isolates, the spacers of these systems did not match any known plasmid or virus sequence.

**Table 2:** **Distribution of Cas-proteins in** ***L. crispatus***

OrthoMCL-family	Cas-model	125-2-CHN	214-1	CTV-05	FB049-03	FB077-07	JV-V01	MV-1A-US	MV-3A-US	SJ-3C-US	ST1	Total	Total with models
LACT01579	Csn2	1/1	1/1	1/1	1/1	1/1	1/1	1/1	1/1	1/1	0	9	9
LACT01697	Cas2	0	1/0	1/1	1/1	1/1	1/1	1/1	1/1	1/0	0	8	6
LACT01698	Cas1	0	1/1	1/1	1/1	1/1	1/1	1/1	1/1	1/1	0	7	7
LACT01822	Csn2	0	1/1	1/1	1/1	1/1	1/1	0	1/1	1/1	0	7	7
LACT01823	Cas9/Csn1	0	1/1	0	1/0	1/0	1/1	1/1	1/1	1/0	0	7	4
LACT01825	Cas9/Csn1	0	0	1/0	1/1	1/1	1/0	1/0	1/0	1/1	0	7	3
LACT02396	Cas9/Csn1	0	0	0	1/1	1/1	0	0	0	1/1	0	3	3
LCRIS_01207	cas-CT1978	0	0	0	0	0	0	0	0	0	1/1	1	1
LCRIS_01209	casE-Cse3	0	0	0	0	0	0	0	0	0	1/1	1	1
LCRIS_01210	casD-Cas5e, CRISPR-cas5	0	0	0	0	0	0	0	0	0	1/1	1	1
LCRIS_01211	casC-Cse4	0	0	0	0	0	0	0	0	0	1/1	1	1
LCRIS_01212	casB-cse2	0	0	0	0	0	0	0	0	0	1/1	1	1
LCRIS_01214	Cas3-core, Cas3-HD	0	0	0	0	0	0	0	0	0	1/1	1	1
	Total	1/1	5/4	5/4	7/6	7/6	6/5	5/4	6/5	7/5	6/6	54	45

The first number in the table cells describes the number of CDSs part of the Cas-protein associated ortholog group in a specific strain and the second number indicates the number of group members matching the Cas-model.

Prompted by the observed differences between the prevalence of CRISPR/Cas systems in the vaginal strains and the chicken isolate, the distribution of the *cas* genes in 135 publicly available *Lactobacillus* genomes was tested (Additional file 7). Markedly, the Type II CRISPR/Cas system hits were more frequent in vaginal (18 of the 40 strains) than in non-vaginal lactobacilli (28 of the 95 strains; Fisher’s exact test *p*-value 0.12), which suggests that the Type II system could be important in the vaginal environment. The prevalence of the other types of CRISPR/cas systems was not significantly different at alpha level 0.20.

### Metabolic pathway reconstruction

Using the automatic annotation server KAAS [59], we were able to assign EC numbers to the members of 1,320 ortholog groups. Surprisingly, the majority of the enzymes belonged to the core groups (Additional file 8), which is somewhat different from the large intra-specific variation present within the metabolic contents of *O. oeni* [80] or *L. paracasei* [38]. In accordance with the high number of core genome-encoded enzymes, the *in silico* reconstruction of *L. crispatus* metabolic pathways suggests that the strains have a potential to utilize a rather same set of carbohydrates (Additional file 9). The data supports the presence of metabolic routes in each strain for the conversion of a variety of sugars into the key intermediates of the pentose phosphate (D-Xylulose 5-phosphate), Embden–Meyerhof–Parnas (D-Fructose 1,6-bisphosphate), and tagatose-6-phosphate (tagatose-6-phosphate) pathways. Pathways for the conversion of the D-Xylulose 5-phosphate and D-Fructose 1,6-bisphosphate into several of their end products were also annotated for nine of the ten strains. The aforementioned indicates the presence of both Embden–Meyerhof–Parnas and pentose phosphate pathways in the nine strains, which is typical for a heterofermentative species and contradictory to the previous classification of *L. crispatus* as a homofermentative species [28, 92]. The exception is the strain CTV-05 that had only partial pathways for many end product conversions, most likely because of sequencing gaps in the corresponding genomic loci. No routes were recorded for the conversion of tagatose-6-phosphate pathway intermediate into pyruvate in any of the strains. Interestingly, the data also shows evidence for the presence of strain-specific glycerone conversions in *L. crispatus* 125-2-CHN.

Regarding urogenital lifestyle, conserved pathways were annotated for the metabolism glucose and mannose, the former reported to be the major free monosaccharide and the latter a minor constituent of the vaginal fluid [93]. Although we did not detect complete routes for the metabolism of glycogen, seven vaginal strains were discovered to carry a gene coding for a type I pullulanase debranching enzyme (LACT01812), which could contribute to the degradation of glycogen. Moreover, *L. crispatus* core appears to encode a sialic acid utilization regulator (RpiR family) and an O-sialoglycoprotein endopeptidase that could contribute to the hydrolysis of O-sialoglycoproteins in the vaginal mucosa. Notably, the manual examination of the enzyme contents revealed that each strain may generate hydrogen peroxide from pyruvate, of which the former acts as an antimicrobial compound.

We also assessed the range of amino acids that *L. crispatus* has a potential to synthetize (Additional file 9). Based on the *in silico* analyses of the biosynthetic capabilities, all strains can synthesize seven amino acids either *de novo* or as derivatives using the same pathways, which is three and four amino acids more than *L helveticus* DPC 4571 [94] or *L. acidophilus* NCFM [84], respectively. Pathways for aspartate biosynthesis were also annotated in nine isolates, excluding the strain CTV-05 that did not share this property. We again speculate that the lack of biosynthesis route for aspartate is rather due to the draft nature of the genome sequence of this strain than a genuine loss. The other differences in amino-acid synthesis related to nuances in synthesis routes for cysteine, serine, and glycine, which seem to vary between isolates. Overall, the *in silico* analyses predicted a dependency on external supplies of amino acids for *L. crispatus* similar to that described for closely related lactobacilli [84, 95] and shows that the strains are rather similar in their biosynthetic power. Moreover, none of the detected conversions was deduced to be strain-specific, further highlighting the similarity.

### Proteinaceous adhesins

Adhesion to host tissue has long been considered an important factor and a prerequisite for the long-term colonization of the human vagina, stimulation of the immune system, and antagonistic activity against harmful pathogens through competitive exclusion [96]. We screened the *L. crispatus* proteomes for adhesion and host colonization related domains and identified 103 proteins governing the ability of *L. crispatus* to colonize and interact with the host. These putative adhesins were associated with seven distinct types of adhesion-associated domains belonging to 21 ortholog groups of which seven are part of the *L. crispatus* core genome (Table 3, Additional file 10). It should be noted, however, that members of the same ortholog group did not necessarily share adhesion domains. In addition, six strain-specific adhesins were identified, all of which were predicted to be mucus-binding proteins. Interesting examples of the strain-specific adhesins include a sortase-anchored protein (LCRIS_00919) with multiple mucus-binding domains, and LCRIS_01654 being the only member of its ortholog group (LACT01522) with adhesion-associated domains. One notable core adhesin (LACT00800) was a putative fibronectin/fibrinogen-binding protein Fbpa, which has recently been proposed to contribute to the fibronectin-binding properties of *Lactobacillus iners* and to explain the stronger adhesion of *L. iners* to human fibronectin compared to other species of *Lactobacillus* tested in the study [97]. Notably, our data does not support this hypothesis, since the presence of functional *fbpa* gene in the *L. crispatus* core genome should have resulted in equal adhesion abilities for the *L. crispatus* and *L. iners* strains tested in the study. Markedly, the recently characterized LEA protein of *L. crispatus* ST1 [33] belonging to LACT00252 was not identified, indicating that this adhesin binds to crop epithelium and epithelial cells from human vagina with some novel domain. In addition to the aforementioned putative adhesins, *L. crispatus* was predicted to harbor ~30 putative S-layer protein-encoding genes that could potentially contribute to bacterial adhesion. However, these predicted S-layer proteins were different from the S-layer proteins of other related lactobacilli reportedly implicated in bacterial adhesion [42, 98, 99].

**Table 3:** **Distribution of adhesion related proteins in** ***L. crispatus***

OrthoMCL- family	Adhesion or colonization domain	125-2-CHN	214-1	CTV-05	FB049-03	FB077-07	JV-V01	MV-1A-US	MV-3A-US	SJ-3C-US	ST1	Total	Total with domain
LACT01267	MucBP	1/1	1/0	1/1	1/1	1/1	1/0	0	1/1	1/1	1/1	9	7
LACT01522	MucBP	1/0	1/0	0	1/0	1/0	1/0	1/0	1/0	1/0	1/1	9	1
LACT01644	MucBP	0	1/1	1/1	1/0	1/0	1/1	1/0	1/1	0	1/1	8	5
LACT01663	MucBP	1/1	1/1	0	1/1	1/1	1/1	1/1	1/1	1/1	0	8	8
LACT01712	MucBP	2/0	0	0	1/1	1/1	1/0	1/0	1/0	1/1	0	7	3
LACT01924	MucBP	1/0	1/1	0	1/1	1/1	1/0	1/0	0	0	0	6	3
LACT02281	MucBP	0	0	0	0	1/1	1/0	1/1	0	0	0	3	2
LACT02327	MucBP	1/0	0	0	0	0	1/1	1/0	0	0	0	3	1
LACT02429	MucBP	2/2	0	0	0	0	0	0	0	0	0	1	1
HMPREF9249_02429	MucBP	0	0	0	0	1/1	0	0	0	0	0	1	1
HMPREF0506_0624	MucBP	0	0	0	0	0	1/1	0	0	0	0	1	1
HMPREF0506_0871	MucBP	0	0	0	0	0	1/1	0	0	0	0	1	1
LCRIS_00919	MucBP	0	0	0	0	0	0	0	0	0	1/1	1	1
LACT00524	Big_3	1/1	1/1	1/1	1/1	1/1	1/1	1/1	1/1	1/1	1/1	10	10
LACT00531	Big_3	1/1	1/1	1/1	1/1	1/1	1/1	1/1	1/1	1/1	1/1	10	10
LACT00945	Big_3	1/0	1/0	1/0	1/1	1/1	1/1	1/1	1/1	1/0	1/1	10	6
LACT02389	Big_3	0	1/1	1/1	0	0	0	0	0	1/1	0	3	3
LACT00169	F5_F8_type_C	1/1	1/1	1/1	1/1	1/1	1/1	1/1	1/0	1/1	1/1	10	9
LACT00800	FbpA, DUF814	1/1	1/1	1/1	1/1	1/1	1/1	1/1	1/1	1/1	1/1	10	10
LACT00237	FIVAR	1/0	1/1	1/1	1/1	1/1	1/1	1/1	1/1	1/1	1/1	10	9
LACT00212	fn3	1/1	1/1	1/1	1/1	1/1	1/1	1/1	1/1	1/1	1/1	10	10
	Total	16/9	13/10	10/9	13/11	15/13	17/12	14/9	12/9	12/10	11/11	131	102

The first number in the table cells describes the number of CDSs part of the ortholog group in a specific strain and the second the number indicates the number of group members having the adhesion or colonization related PFAM-domain(s).

### Cell wall exopolysaccharide

**Figure 4:**
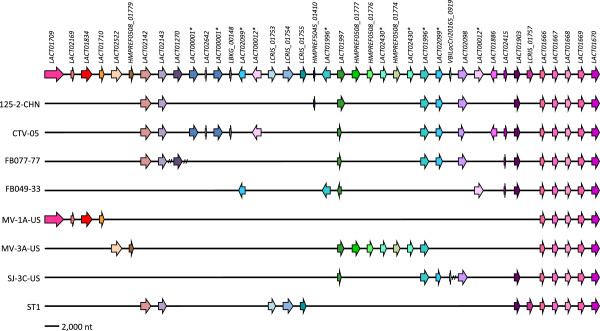
**Variation in EPS gene cluster in** ***L. crispatus*** **.** The organization and conservation of the exopolysaccharide synthesis regions in *L. crispatus.* Orthologous genes are represented with the same color and stars indicate genes found in different loci. Dashed lines represent contig breaks in the MV-1A-US and SJ-3C-US clusters.

In the *L. crispatus* genomes, a highly variable genome region appears to be associated with EPS biosynthesis. This EPS gene cluster was observed in eight *L. crispatus* strains and noted to comprise 37 EPS biosynthesis genes, five of which were present within each operon (Figure 4). The five conserved genes were predicted to encode a transcriptional regulator, a polymerization and chain length determination protein, a tyrosine-protein kinase, a protein-tyrosine phosphatase, and the priming glycosyltransferase. The remaining genes coded for proteins with putative glycosyl transferase functions, indicating that the strains produce EPSs with different sugar monomers and glycosidic linkages. Markedly, EPS gene clusters were not detected in the genomes of *L. crispatus* JV-V01 and 214-1.

### Antimicrobial potential in *L. crispatus*

*Lactobacillus* species can maintain the vaginal ecosystem in a healthy condition by the production of antimicrobial substances such as lactic acid, hydrogen peroxide and bacteriocin-like substances [9, 96]. Lactic acid is the main end product of the carbohydrate fermentation in lactobacilli and can contribute to the vaginal acidity and thereby inhibit the colonization and proliferation of harmful micro-organisms in the vagina [100]. The *L. crispatus* strains studied here appeared to possess between three to four L-lactate dehydrogenases for the conversion of puryvate into lactic acid. Interestingly, one specific *ldh* locus found in five *L. crispatus* strains was flanked by a transposase enzyme gene that may affect its expression [101]. We also discovered hydrogen peroxide producing enzymes (EC:1.2.3.3 and EC:1.1.3.15) in each *L. crispatus*, which correlates well with the experimental data showing that hydrogen peroxide generation is common among vaginal *L. crispatus* [102].

Using BAGEL [63], the bacteriocin content of *L. crispatus* was investigated (Table 4). This method was able classify several sets of putative bacteriocin gene clusters in each strain, including at least two regions encoding bacteriolysins (similar to enterolysin A [103] and helveticin J [104]). In addition, regions implicated in the production of class II bacteriocins were revealed in the vaginal isolates. A pediocin-like bacteriocin that inhibits the growth of pathogenic *Listeria* and *Clostridium* species [105] was present in five vaginal isolates and all nine encoded a two-component bacteriocin LS2 that inhibits the growth of isolates belonging to genera *Listeria*, *Shigella,* and *Yersinia* [106]. Notably, the pediocin-like bacteriocin encoding genes were found in the vicinity of CDSs encoding proteins harboring a domain for Enterocin A immunity.

**Table 4:** **Distribution of predicted bacteriocin related proteins in** ***L. crispatus***

Bacteriocin class	Bacteriocin type	PFAM	125-2-CHN	214-1	CTV-05	FB049-03	FB077-07	JV-V01	MV-1A-US	MV-3A-US	SJ-3C-US	ST1	Total	Total with domain
Bacteriocins larger than 10kD, Class III	Enterolysin A	Peptidase_M23	1/1	2/2	2/2	2/2	2/2	2/2	2/2	2/2	2/2	2/2	10	10
	Bacteriocin helveticin J	-	1	1	1	1	1	1	1	1	1	1	10	-
	Helveticin J	-	1	1	1	1	2	1	1	1	1	1	10	-
LAPs	Small orfs	-	1	0	0	1	1	0	1	0	0	0	4	-
Small unmodified bacteriocins, Class II	Penocin A	Bacteriocin_II	1/2	0	1/2	1/2	1/2	0	0	1/2	1/1	0	6	6
	LS2, chain A	Bacteriocin_IIc	1/1	1/1	1/1	1/1	1/1	1/1	1/1	1/1	1/1	0	9	9
		Total	7	5	7	8	9	5	6	7	6	4	49	25

For the bacteriocin-like molecules with PFAM-domain, the first number in the table cells describes the number of CDSs part of the ortholog group in a specific strain, and the second number indicates the number of group members having the PFAM-domain. Helveticin J refers to the antimicrobial molecule first identified in *L. helveticus* and bacteriocin helveticin J to one identified in *L. acidophilus*.

### Antagonistic activities against *G. vaginalis*

BV is the most common vaginal disorders, affecting up to a third of women [107]. It has been associated with increased risk for preterm birth, urinary tract infections, and HIV infection, and represents a condition in which the normal protective lactobacilli community is replaced by an overgrowth of anaerobic bacteria [46]. Although the etiology of BV is not known, *G. vaginalis* is present in up to 95% of all BV cases [108], indicating that it could have a role in BV. In our efforts to decipher the genetic basis of the inhibitory actions of the species *L. crispatus* against *G. vaginalis,* we performed ortholog grouping of the available *G. vaginalis* data (Additional file 11) and used comparative genomics to identify shared common molecular mechanisms between *G. vaginalis* and *L. crispatus*. Importantly, our analyses revealed several components by which *L. crispatus* could interfere with the attachment of *G. vaginalis* in the vagina. Firstly, fibronectin-binding could play a role in this process, given that proteins with FIVAR domains related to hyaluronate or fibronectin-binding were encoded in the core genomes of both *G. vaginalis* (GVAG00006) and *L. crispatus* (LACT00237). Secondly, searching *L. crispatus* proteins against the *G. vaginalis* HMM database suggested another *L. crispatus* protein (LACT01268), which could play a role in preventing the cell adhesion of *G. vaginalis* to fibronectin. Intriguingly, this counterpart of the *G. vaginalis* FIVAR-proteins was distributed in nine *L. crispatus* strains, but had no known adhesion domains. Another interesting core orholog group of *G. vaginalis* was GVAG00055. Many members of this ortholog group contained a bacterial Ig-like domain (PF12245), which is distantly related to the interaction domains, namely fn3 (PF00041) and Big_3 (PF07523), associated with several *L. crispatus* core adhesins (Table 3). Moreover, searches against the *G. vaginalis* HMMs revealed two additional *L. crispatus* adhesins (LACT01712 and LACT02327) that could act as counterparts of GVAG00055, although having mucin-binding domains (Table 3). Finally, of the three *G. vaginalis* pilus-encoding gene clusters that were identified based on the pilus-encoding genes listed by Yeoman *et al.* [72], the one associated with most isolates had borderline (E-value ≤ 0.4) counterparts in the *L. crispatus* core genome. Its major subunit pilin (GVAG00005) appears to have two potential antagonists in the *L. crispatus* core genome encoding a 12.8-kilodalton protein (LACT00214) and the LEA protein (LACT00252). In addition, the long CDS (GVAG00017) located next to the major subunit component in the cluster and showing similarity to known adhesins and surface antigens, could be inhibited by the members of the LACT01712 and LACT02440 based on the *G. vaginalis* HMM searches. Taken together, these findings indicate that *L. crispatus* could interfere with fibronectin-binding and pilus components of *G. vaginalis*.

Of the other listed virulence-related factors in *G. vaginalis* [72], the invasion-associated hydrolase (GVAG00614), protein with two G-related albumin-binding modules (GVAG01097), NLPA lipoprotein (GVAG00181), and endothelin-converting enzyme (GVAG00141) have potential antagonists encoded by the *L. crispatus* core based on the *G. vaginalis* HMM searches. A noteworthy finding is that the G-related albumin-binding module protein (GVAG01097) present in 17 *G. vaginalis* isolates shared similarity with 42 *L. crispatus* proteins, including all nine FIVAR-domain associated proteins of the LACT00237 (Table 3).

### Adhesion inhibition assays to HeLa cells

**Figure 5:**
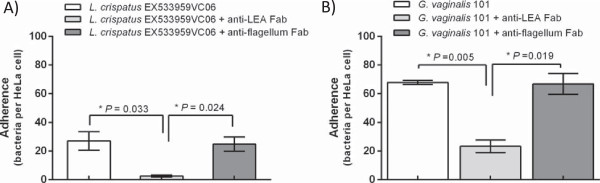
**Inhibition of** ***L. crispatus*** **or** ***G. vaginalis*** **adhesion to HeLa cells by LEA-specific Fab fragments.** Cells of *L. crispatus* EX533959VC06 **(A)** or *G. vaginalis* 101 **(B)** were pretreated with LEA-specific IgG Fab fragments or unrelated anti-flagellum Fab fragments or left untreated in PBS supplemented with 5 mM PMSF before the adhesion assays. The number of adherent bacteria per epithelial cell in 20 randomly chosen microscopic fields was determined. The assay was performed twice with duplicate samples and the results show mean values of adherent bacteria. The asterisk indicates P < 0.05 as calculated by Student’s *t* test.

Our comparative analysis described several species-wide factors by which *L. crispatus* could compete with *G. vaginalis* in the vagina. For example, the LEA protein was identified as a prominent counterpart of one of the *G. vaginalis* core adhesins and was thereby predicted to participate in the adherence inhibition of this pathogen. To validate the role of LEA in the antagonism against *G. vaginalis*, the adhesion capacity of a vaginal *L. crispatus* isolate EX533959VC06 and BV-associated *G. vaginalis* 101 to HeLa cells was tested using the previously described approach [17] with and without the pretreatment with Fab fragments prepared against LEA [33]. Markedly, the anti-LEA Fab fragments significantly reduced the adhesion level of both bacterial species to HeLa cells whereas the unrelated anti-flagellum Fab fragments showed no inhibitory effect (Figure 5). The reduction in adherence was most evident for the strain EX533959VC06; the anti-LEA Fab fragment pretreatment resulting in 90.6% (*p*-value ≤ 0.033) and 89.8% (*p*-value ≤ 0.024) reduction in adhesion to HeLa cells compared with the untreated or anti-flagellum Fab fragment pretreated bacteria, respectively. Intriguingly, pretreating *G. vaginalis* 101 with the anti-LEA Fab fragments caused also a significant reduction in adherence compared with the untreated bacterial cells (65.6%; *p*-value ≤ 0.005) or bacteria pretreated with the control anti-flagellum Fab fragments (65.1%; *p*-value ≤ 0.019). These observations validated the predicted competitive character between LEA and *G. vaginalis*, suggesting a role for LEA in the previously identified ability of *L. crispatus* to exclude and displace *G. vaginalis* from HeLa cells [17]. The results also provide an explanation to the inverse association between *L. crispatus* and *G. vaginalis* colonization in the vagina [12, 44, 47]. Based on our comparative genomic analyses, the LEA protein achieves its inhibitory effect by competing with the same attachment sites as the pili of *G. vaginalis*. Of note, our adhesion assay provided a further support for the species-wide distribution of LEA among *L. crispatus*, since the strain EX533959VC06 has not yet been sequenced. Furthermore, since LEA has previously been studied only in the chicken isolate ST1 [33], our results serve as the first record of the functionality of LEA in vaginal *L. crispatus.*

### Phylogenetic relations

**Figure 6:**
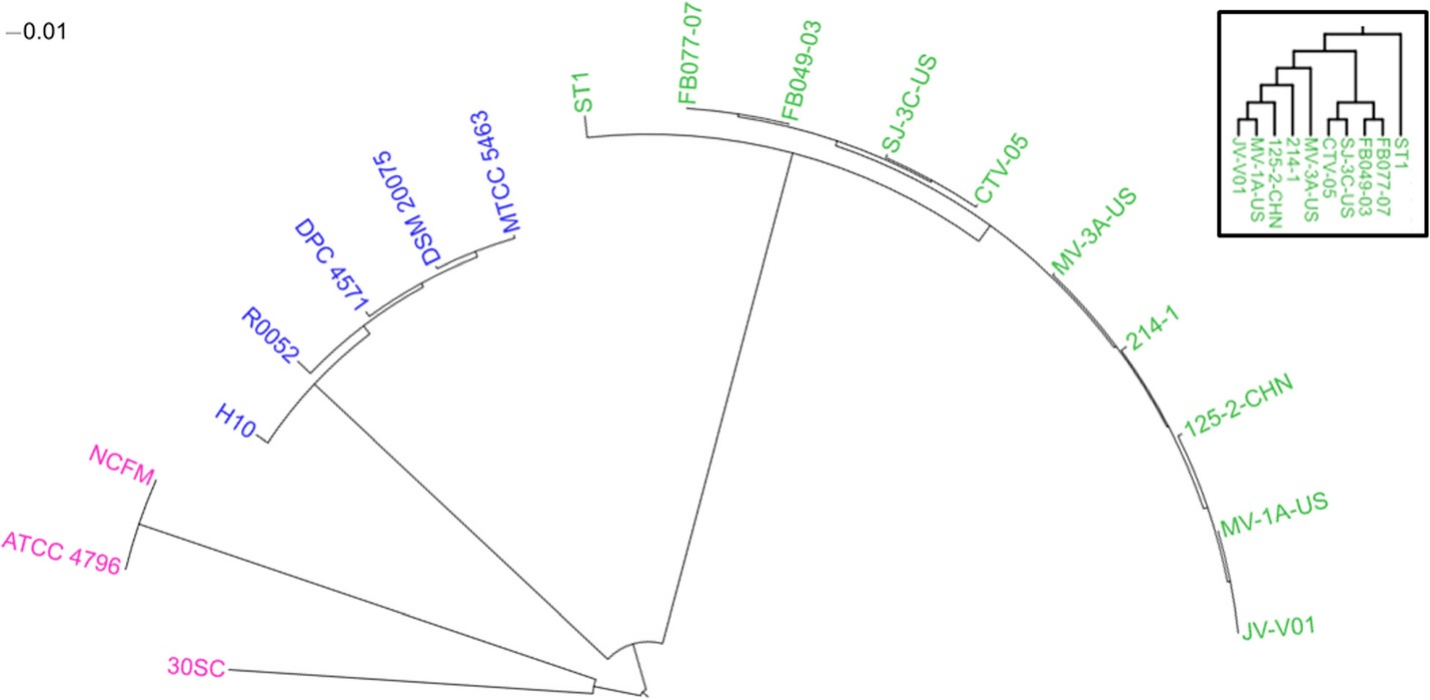
**Phylogenetic tree.** Phylogenetic relations of the selected *L. crispatus* (green), *L. helveticus* (blue) and *L. acidophilus* (purple) strains based on the SNPs of the core genome. The *B. subtilis* genome was used as the out-group to root the tree, but is not shown in the figure. In the inset, the branching pattern of the *L. crispatus* strains is highlighted.

Phylogentic relations between the selected *L. crispatus* strains and strains of closely related species *L. acidophilus* and *L. helveticus* were examined based on a maximum-likelihood tree built from the SNPs of the core genome. Altogether 38,726 conserved polymorphic sites were identified from the genome alignments and used for the construction of a phylogenetic tree. The phylogenetic tree (Figure 6) clearly shows that strains of the same species cluster together and that each *Lactobacillus* species has differentiated as a distinct entity. The species *L. crispatus* and *L. helveticus* share the most recent common ancestor and form a sister group to species *L. acidophilus*, which is accordance with previously reported phylogenetic trees [29, 109]. Among the *L. crispatus* cluster, the chicken isolated ST1 branches off first from the vaginal isolates.

## Conclusions

The rapidly increasing number of complete microbial genomes offers previously unimaginable possibilities to understand the phenotypic and genomic diversity in a particular species [38, 70, 79, 80]. In this study, we have taken advantage of publicly available *L. crispatus* genomes and present the genetic landscape of this important urogenital lactic acid bacterium [7–12]. We assessed the overall genomic similarity of ten strains and defined the *L. crispatus* pan- and core genomes. These analyses depicted high sequence identity and extensive synteny punctuated by several GIs, and revealed a current pan-genome that is nearly two times larger than the number of ortholog groups present in an average *L. crispatus* strain. About one third of all 3,929 ortholog groups were assigned to all strains, constituting the current *L. crispatus* core genome and encoding the basic aspects of *L. crispatus* biology. Importantly, these core features comprised several CDSs for the production of antimicrobial molecules and competitive exclusion of the BV associated species *G. vaginalis*, shedding light on the molecular mechanisms by which *L. crispatus* could maintain vaginal health*.* The pan-genome analysis also revealed 1,311 singleton ortholog groups associated with only one strain. The enrichment of functions related to replication and repair among these genes indicates the influence of transposons in genome evolution in this species. A third of the strain-specific ortholog groups had the highest similarity to genes found in the other strains of the *L. delbrueckii* clade, suggesting notable sequence influx from closely related lactobacilli. Our regression analysis indicates that the genetic diversity present within *L. crispatus* has not yet been comprehensively captured. Specifically, we estimate that over ten new ortholog groups will be discovered per every additional genome until almost 300 *L. crispatus* strains have had their genomes defined. This estimation may be compromised by the uncertainty caused by the draft genomes that have up to 201 sequence gaps. Nevertheless, the data implies the presence of large repertoires of undiscovered *L. crispatus* genes to be sequenced in the future. The phylogenetic tree based on core genome SNPs among the ten isolates revealed that the chicken isolated ST1 branches off first from the *L. crispatus* cluster and that the *L. acidophilus* cluster is a sister taxon to *L. helveticus* and *L. crispatus*, as suggested earlier [29, 109].

From the perspective of vaginal health, the most interesting genomic diversity regions in *L. crispatus* include the loci related to EPS biosynthesis, prophages and adaptive immunity, of which the latter two may play a role in BV. Firstly, the genetic differences in the composition of the EPS gene region may participate in the *L. crispatus* adhesion, biofilm formation and competitive exclusion of pathogens. The EPS-deficient strains JV-V01 and 214-1 are particularly interesting, as the deprivation of EPS has been reported to promote bacterial adhesion in other lactobacilli [110, 111]. Secondly, the presence of prophage-like clusters in the vaginal *L. crispatus* genomes is in accordance with the previously observed [85] high level of lysogeny in vaginal *L. crispatus* strains. If truly inducible, the spontaneous release of the prophages could contribute to the development of BV [86]. Finally, a relationship was depicted between the life environment of the strains and their adaptive immunity systems, suggesting that different types CRISPR/Cas systems could be beneficial in different environments. This hypothesis is further supported by the analysis of the *cas* gene contents of 135 *Lactobacillus* genomes that revealed higher rates of the Type II CRISPR/Cas systems in vaginal than in non-vaginal lactobacilli. In addition, the CRISPR-arrays of the vaginal *L. crispatus* strains carry evidence of encounters with common invaders, as several of the spacer sequences were identical between several strains.

The defined *L. crispatus* core genome helps to explain how this species can thrive in the vaginal environment and benefit vaginal health. In the vaginal epithelium of reproductive age females, large quantities of glycogen are broken down and then metabolized into lactic acid, which is thought to result in acidification of the vagina [112, 113]. Although *L. crispatus* lacks complete enzymatic machinery for glycogen degradation, the core genome encodes enzymatic pathways for the utilization of a range of carbohydrates available in the vaginal fluid, which could support the urogenital commensal lifestyle of *L. crispatus.* Encoded in the core are also several features potentially governing host-interactions and displaying an antagonistic activity against other micro-organisms. Interestingly, the bacteriocin-like molecules encoded by the *L. crispatus* genomes could inhibit biofilm integrated *G. vaginalis* cells*,* shown to be more resistant to hydrogen peroxide and lactic acid than the cells in planktonic state [114]. Specifically, as *G. vaginalis* is known to develop an adherent biofilm on the vaginal epithelium in BV [115] this property could provide attractive means to restore the normal vaginal flora. In addition to the antimicrobial properties, *L. crispatus* was detected to contain several proteins that could mediate the previously reported [17] competitive exclusion of *G. vaginalis* from epithelial cells and explain the inverse association between *L. crispatus* and *G. vaginalis* colonization in the vagina [12, 44, 47] *.* Most notably, these specific interference mechanisms might include blocking the attachment of *G. vaginalis* by disturbing the pilus-mediated adhesion of the pathogen. This mechanism could involve LEA, shown here to be universally present in all *L. crispatus* strains, and demonstrated using LEA-specific Fab fragments to inhibit the adhesion of *G. vaginalis* adhesion to HeLa cells*.* Although LEA showed sequence similarity to a pilus component of *G. vaginalis*, further studies are still needed to decipher whether the counterpart of LEA is indeed the pilin subunit or some other adhesion associated molecule of *G. vaginalis.* In addition, we cannot rule out that surface molecules other than the ones recognized by the anti-LEA Fab fragments have participated in the contact between *G. vaginalis* and the host cell, since the Fab fragments did not abolish the adhesion completely. Nevertheless, the LEA protein appears to be a key mediator of the competitive exclusion of *G. vaginalis.*

In summary, we have presented a comparative analysis of ten *L. crispatus* genomes available within the public databases at the time of this study and provided a comprehensive look on the pan-genomic structure of this important urogenital species. Furthermore, our analyses revealed a list of core genes implicated in protecting the urogenital tract from *G. vaginalis* colonization, providing new insights into the treatment and prevention of BV.

## Electronic supplementary material

Additional file 1: **Overview of** ***G. vaginalis*** **strains and properties.** In the table, the genomic properties of the *G. vaginalis* strains used in this study are given. HMP refers to the Human Microbiome Project. (PDF 103 KB)

Additional file 2: **List of** ***L. helveticus, L. acidophilus*** **and** ***B. subtilis*** **genomes included in the phylogenetic analysis.** The accession is given for each genome. (PDF 41 KB)

Additional file 3: **List of PFAM domains used in the annotation of putative** ***L. crispatus*** **adhesins.** The accession, ID, and description are given for each PFAM matching an adhesion or colonization related keyword. Ones in the remaining columns indicate that the PFAM matched some *L. crispatus* CDS, passed the manual curation process and was included in the final list of adhesion or colonization related domains. (XLS 180 KB)

Additional file 4: **The start and end compounds used in the metabolism screens.** This table describes the compound pairs related to the *de novo* synthesis and interconversion of amino acids and carbohydrate metabolism. (XLS 74 KB)

Additional file 5: **The** ***L. crispatus*** **data table.** Results of the different bioinformatic analyses for each *L. crispatus* CDS. (XLS 18 MB)

Additional file 6: **Reservoirs of genetic variability.** This table describes the distribution of best BLAST hits of strain-specific *L. crispatus* CDSs. (XLS 28 KB)

Additional file 7: **Prevalence of different types of CRISPR/Cas systems in vaginal and non-vaginal lactobacilli.** (XLS 20 KB)

Additional file 8: **Variation in metabolism related enzymes in** ***L. crispatus.*** The horizontal lines represent the orthologous groups with assigned EC numbers and the different strains are indicated at the bottom of the picture. The presence of a given ortholog group in a specific strain is indicated with grey (single copy) or dark grey (duplicated genes). The absence of a given ortholog group is indicated with light grey. The colored bar on the left describes the conservation level of the ortholog groups and follows that of the Figure 2 (red indicates core genome and blue accessory genome with darkest shade indicating conservation in nine strains and lightest blue strain-specific). The dendrogram was generated using ward linkage clustering of the presence/absence data. (PDF 72 KB)

Additional file 9: **Detected metabolic routes between different carbohydrate metabolism compounds and between different amino acid biosynthesis related compounds.** The presence of intact metabolic route from one compound to another is indicated for each *L. crispatus* genome. Pathways found in the core genome analysis are marked with yellow color. (XLS 44 KB)

Additional file 10: **Domain organization of** ***L. crispatus*** **adhesion and colonization factors.** A representative member of each OrthoMCL-group is presented graphically. The larger colored blocks represent adhesion or colonization related PFAM-domains and the thinner blocks other domains. The names of each color-coded domain are given at the bottom of the picture. (PDF 145 KB)

Additional file 11: **Overview of** ***G. vaginalis*** **ortholog groups.** For each group, the associated proteins, PFAM domains, matching proteins in *L. crispatus,* the total number of matching proteins in *L. crispatus*, and total number of *L. crispatus* genomes providing the matches are given in the table. (XLS 3 MB)

## Abbreviations

BV: Bacterial vaginosis
Cas: CRISPR-associated
CDS: Coding sequence
CFUs: Colony forming units
COG: Cluster of orthologous groups
CRISPR: Clustered regularly interspaced short palindromic repeat
EPS: Exopolysaccharide
GI: Genomic Island
GIT: Gastrointestinal tract
GUT: Genitourinary tract
HGT: Horizontal gene transfer
HMM: Hidden Markov model
LEA: *Lactobacillus* epithelium adhesin
OD: Optical density
PBS: Phosphate buffered saline
PFA: Paraformaldehyde
PMSF: Phenylmethylsulfonyl fluoride
RUTI: Recurrent urinary tract infection
SNP: Single nucleotide polymorphism.
