# Supplementary material for: Comparative genomics of Lactobacillus crispatus suggests novel mechanisms for the competitive exclusion of Gardnerella vaginalis
Source: BMC Genomics. 2014 Dec 5;15:1070. doi: 10.1186/1471-2164-15-1070 (PMC4300991; doi:10.1186/1471-2164-15-1070)
Supplement: Supplementary file 1 — Additional file 1: Overview of G. vaginalis strains and properties. In the table, the genomic properties of the G. vaginalis strains used in this study are given. HMP refers to the Human Microbiome Project. (PDF 103 KB) [file 12864_2014_6771_MOESM1_ESM.pdf]

Strain	Accession	Scaffolds	Genome size (Mb)	CDS	Average CDS length	Comments	Reference
00703Bmash	[GenBank:ADET00000000]	16	1.57	1256	1083	isolated from a BV patient undergoing metronidazole treatment ^	Ahmed A *et al*. 2012
00703C2mash	[GenBank:ADEU00000000]	22	1.55	1236	1080	isolated from a BV patient undergoing metronidazole treatment ^	Ahmed A *et al*. 2012
00703Dmash	[GenBank:ADEV00000000]	11	1.49	1166	1123	isolated from a BV patient undergoing metronidazole treatment ^	Ahmed A *et al*. 2012
0288E	[GenBank:ADEN00000000]	17	1.71	1356	1093	clinical isolate, biotype 1	Ahmed A *et al*. 2012
1400E	[GenBank:ADER00000000]	28	1.72	1383	1068	clinical isolate, biotype 4	Ahmed A *et al*. 2012
1500E	[GenBank:ADES00000000]	27	1.55	1215	1109	clinical isolate, biotype 5	Ahmed A *et al*. 2012
284V	[GenBank:ADEL00000000]	16	1.65	1285	1111	clinical isolate, biotype 1	Ahmed A *et al*. 2012
315-A	[GenBank:AFDI00000000]	13	1.65	1290	1116	clinical isolate	Nelson KE *et a*l. 2010 (HMP)
409-05	[GenBank:NC_013721.1]	1	1.62	1258	1089	isolated from the vagina of a healthy individual	Nelson KE *et al.* 2010 (HMP), Yeoman CJ *et al*. 2010
5.Jan	[GenBank:ADAN00000000]	94	1.67	1335	1054	isolated from a healthy vagina	Harwich MD Jr *et al*. 2010
55152	[GenBank:ADEQ00000000]	25	1.64	1298	1075	clinical isolate, biotype 3	Ahmed A *et al*. 2012
6119V5	[GenBank:ADEW00000000]	12	1.50	1172	1120	isolated from the vagina of an asymptomatic patient, biotype 7	Ahmed A *et al*. 2012
6420B	[GenBank:ADEP00000000]	14	1.49	1142	1134	isolated from the vagina of an asymptomatic patient, biotype 2 *	Ahmed A *et al*. 2012

6420LIT	[GenBank:ADEO00000000]	3	0.54	369	1294	isolated from the vagina of an asymptomatic patient, biotype 2 *	Ahmed A *et al*. 2012
75712	[GenBank:ADEM00000000]	3	1.67	1312	1100	isolated from the vagina of an asymptomatic patient, biotype 1	Ahmed A *et al*. 2012
AMD	[GenBank:ADAM00000000]	117	1.61	1313	1043	isolated from the vagina of a woman with BV	Harwich MD Jr *et al*. 2010
ATCC14019	[GenBank:NC_014644.1]	1	1.67	1365	1066	isolated from the vaginal secretions of a BV patient	Nelson KE *et al*. 2010 (HMP), Yeoman CJ *et al*. 2010
HMP9231	[GenBank:NC_017456.1]	1	1.73	1317	1106	isolated from the oral cavity	Nelson KE *et al*. 2010 (HMP)
JCP7275	[GenBank:ATJS00000000]	202	1.56	1428	971	vaginal isolate	Nelson KE *et al*. 2010 (HMP)
JCP7276	[GenBank:ATJR00000000]	179	1.66	1486	993	vaginal isolate	Nelson KE *et al*. 2010 (HMP)
JCP7659	[GenBank:ATJQ00000000]	214	1.53	1439	950	vaginal isolate	Nelson KE *et al*. 2010 (HMP)
JCP7672	[GenBank:ATJP00000000]	169	1.60	1396	1021	vaginal isolate	Nelson KE *et al*. 2010 (HMP)
JCP7719	[GenBank:ATJO00000000]	185	1.56	1485	935	vaginal isolate	Nelson KE *et al*. 2010 (HMP)
JCP8017A	[GenBank:ATJN00000000]	187	1.61	1515	944	vaginal isolate	Nelson KE *et al*. 2010 (HMP)
JCP8017B	[GenBank:ATJM00000000]	187	1.60	1517	941	vaginal isolate	Nelson KE *et al*. 2010 (HMP)
JCP8066	[GenBank:ATJL00000000]	197	1.52	1407	961	vaginal isolate	Nelson KE *et al*. 2010 (HMP)
JCP8070	[GenBank:ATJK00000000]	173	1.48	1366	963	vaginal isolate	Nelson KE *et al*. 2010

							(HMP)
JCP8108	[GenBank:ATJJ00000000]	176	1.66	1516	977	vaginal isolate	Nelson KE *et al*. 2010 (HMP)
JCP8151A	[GenBank:ATJI00000000]	189	1.56	1452	955	vaginal isolate	Nelson KE *et al*. 2010 (HMP)
JCP8151B	[GenBank:ATJH00000000]	185	1.55	1459	952	vaginal isolate	Nelson KE *et al*. 2010 (HMP)
JCP8481A	[GenBank:ATJG00000000]	204	1.57	1482	926	vaginal isolate	Nelson KE *et al*. 2010 (HMP)
JCP8481B	[GenBank:ATJF00000000]	180	1.57	1431	958	vaginal isolate	Nelson KE *et al*. 2010 (HMP)
JCP8522	[GenBank:ATJE00000000]	191	1.47	1365	964	vaginal isolate	Nelson KE *et al*. 2010 (HMP)

* isolated from the same patient

^ isolated from the same patient

REFERENCES

Ahmed A, Earl J, Retchless A, Hillier SL, Rabe LK, Cherpes TL, Powell E, Janto B, Eutsey R, Hiller NL, Boissy R, Dahlgren ME, Hall BG, Costerton JW, Post JC, Hu FZ, Ehrlich GD: Comparative genomic analyses of 17 clinical isolates of Gardnerella vaginalis provide evidence of multiple genetically isolated clades consistent with subspeciation into genovars. J Bacteriol. 2012, 94(15):3922-37.

Harwich MD Jr, Alves JM, Buck GA, Strauss JF, Patterson JL, Oki AT, Girerd PH, Jefferson KK: Drawing the line between commensal and pathogenic Gardnerella vaginalis through genome analysis and virulence studies. BMC Genomics 2010, 11:375.

Nelson KE, Weinstock GM, Highlander SK, Worley KC, Creasy HH, Wortman JR, Rusch DB, Mitreva M, Sodergren E, Chinwalla AT, Feldgarden M, Gevers D, Haas BJ, Madupu R, Ward DV, Birren BW, Gibbs RA, Methe B, Petrosino JF, Strausberg RL, Sutton GG, White OR, Wilson RK, Durkin S, Giglio MG, Gujja S, Howarth C, Kodira CD, Kyrpides N, Mehta T, Muzny DM, Pearson M, Pepin K, Pati A, Qin X, Yandava C, Zeng Q, Zhang L, Berlin AM, Chen L, Hepburn TA, Johnson J, McCorrison J, Miller J, Minx P, Nusbaum C, Russ C, Sykes SM, Tomlinson CM, Young S, Warren WC, Badger J, Crabtree J, Markowitz VM, Orvis J, Cree A, Ferriera S, Fulton LL, Fulton RS, Gillis M, Hemphill LD, Joshi V, Kovar C, Torralba M, Wetterstrand KA, Abouellleil A, Wollam AM, Buhay CJ, Ding Y, Dugan S, FitzGerald MG, Holder M, Hostetler J, Clifton SW, Allen-Vercoe E, Earl AM, Farmer CN, Liolios K, Surette MG, Xu Q, Pohl C, Wilczek-Boney K, Zhu D: A catalog of reference genomes from the human microbiome. Science 2010, 328(5981):994-999.

Yeoman CJ, Yildirim S, Thomas SM, Durkin AS, Torralba M, Sutton G, Buhay CJ, Ding Y, Dugan-Rocha SP, Muzny DM, Qin X, Gibbs RA, Leigh SR, Stumpf R, White BA, Highlander SK, Nelson KE, Wilson BA: Comparative genomics of Gardnerella vaginalis strains reveals substantial differences in metabolic and virulence potential. PLoS One. 2010, 26;5(8):e12411.
