# Supplementary material for: Comparative genomics of Lactobacillus crispatus suggests novel mechanisms for the competitive exclusion of Gardnerella vaginalis
Source: BMC Genomics. 2014 Dec 5;15:1070. doi: 10.1186/1471-2164-15-1070 (PMC4300991; doi:10.1186/1471-2164-15-1070)
Supplement: Supplementary file 2 — Additional file 2: List of L. helveticus, L. acidophilus and B. subtilis genomes included in the phylogenetic analysis. The accession is given for each genome. (PDF 41 KB) [file 12864_2014_6771_MOESM2_ESM.pdf]

Species	Strain	Accession
Lactobacillus acidophilus	30SC	[GenBank:NC_015214]
Lactobacillus acidophilus	ATCC 4796	[GenBank:GG669567]
Lactobacillus acidophilus	NCFM	[GenBank:NC_006814]
Lactobacillus helveticus	R0052	[GenBank:NC_018528]
Lactobacillus helveticus	H10	[GenBank:NC_017467]
Lactobacillus helveticus	DPC 4571	[GenBank:NC_010080]
Lactobacillus helveticus	DSM 20075	[GenBank:GG700753]
Lactobacillus helveticus	MTCC 5463	[GenBank:AEYL01000001]
Bacillus subtilis	168	[GenBank:NC_000964]
