# Supplementary figures and images for: Comparative genomics of Lactobacillus crispatus suggests novel mechanisms for the competitive exclusion of Gardnerella vaginalis
Source: BMC Genomics. 2014 Dec 5;15:1070. doi: 10.1186/1471-2164-15-1070 (PMC4300991; doi:10.1186/1471-2164-15-1070)

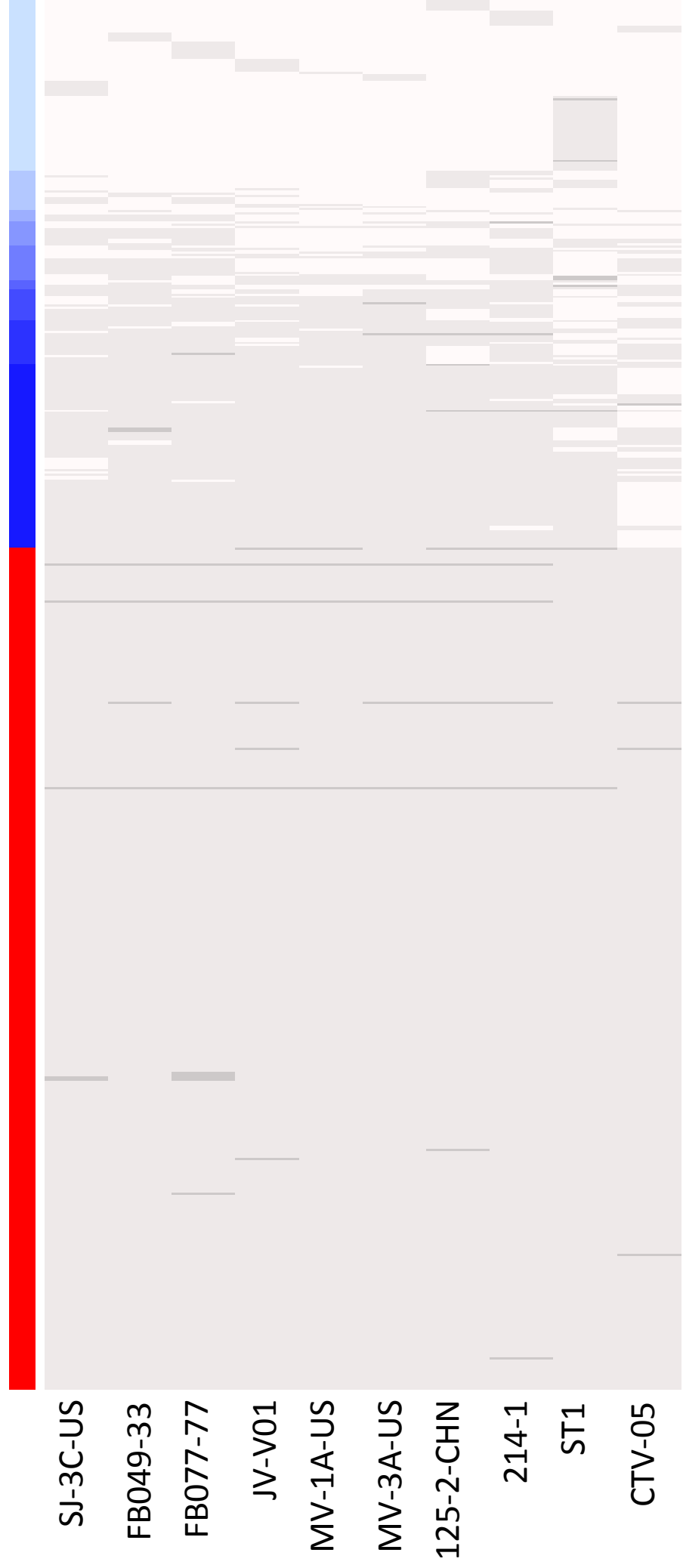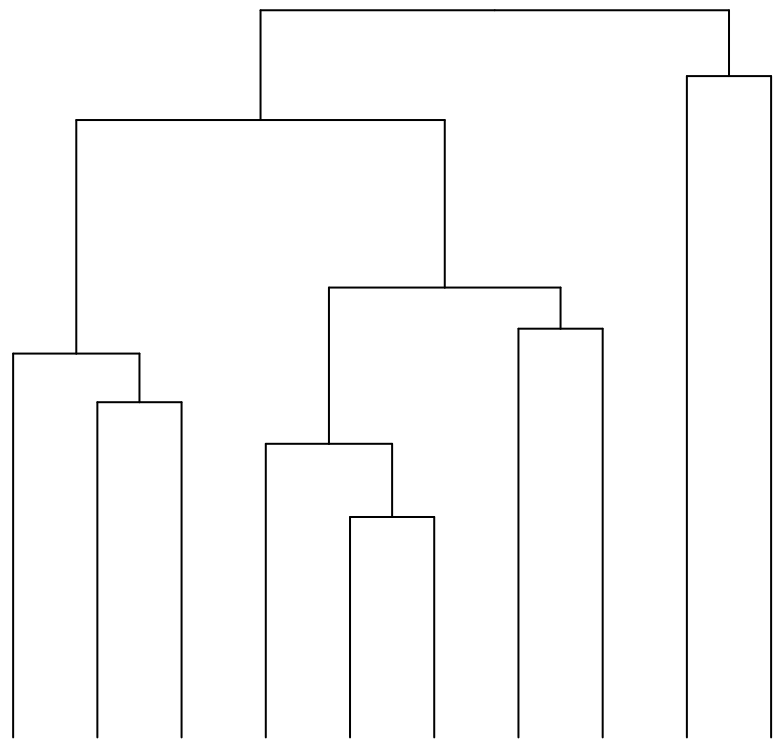

Supplement: Supplementary file 8 — Additional file 8: Variation in metabolism related enzymes in L. crispatus. The horizontal lines represent the orthologous groups with assigned EC numbers and the different strains are indicated at the bottom of the picture. The presence of a given ortholog group in a specific strain is indicated with grey (single copy) or dark grey (duplicated genes). The absence of a given ortholog group is indicated with light grey. The colored bar on the left describes the conservation level of the ortholog groups and follows that of the Figure 2 (red indicates core genome and blue accessory genome with darkest shade indicating conservation in nine strains and lightest blue strain-specific). The dendrogram was generated using ward linkage clustering of the presence/absence data. (PDF 72 KB) [file 12864_2014_6771_MOESM8_ESM.pdf]

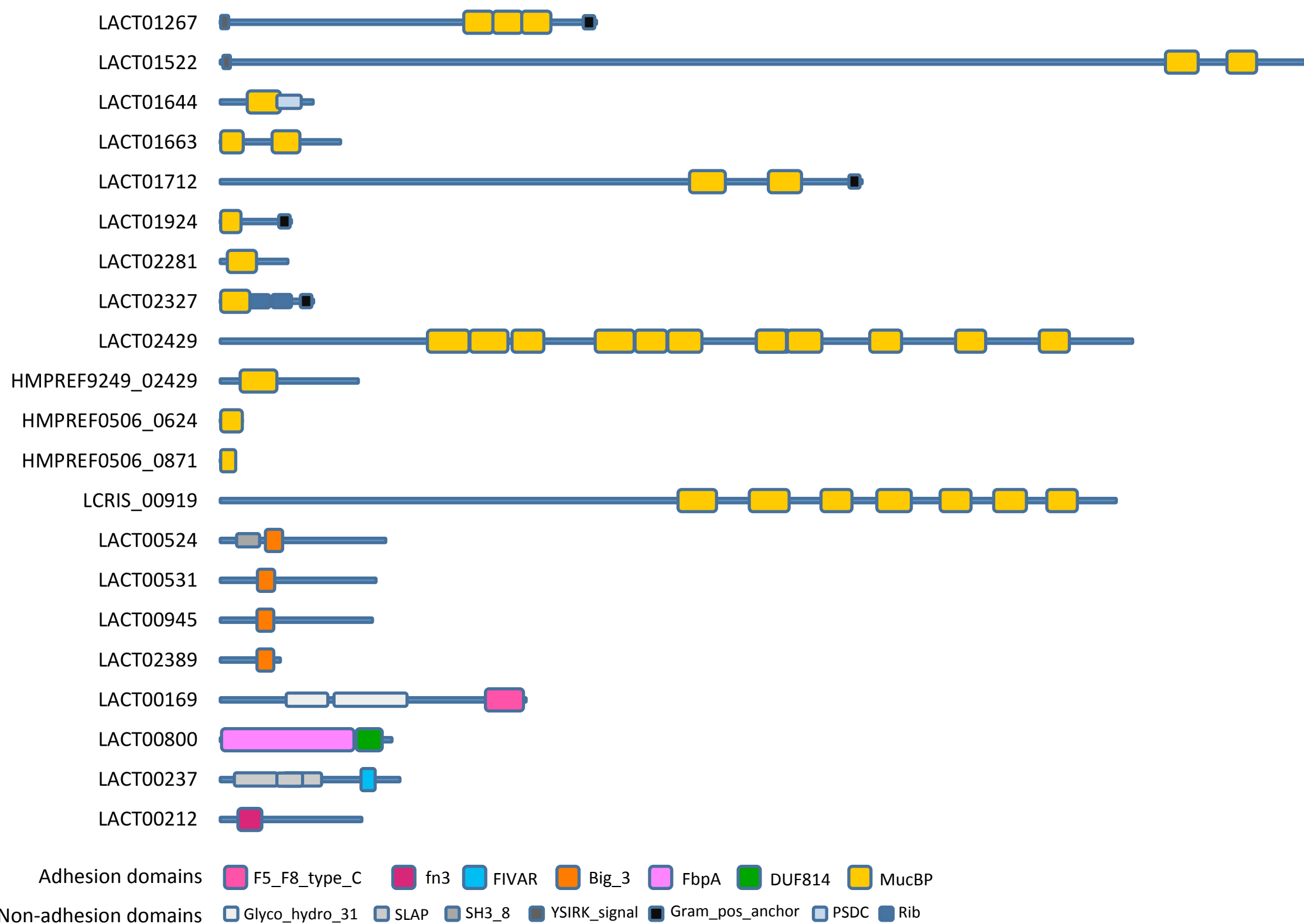

Supplement: Supplementary file 10 — Additional file 10: Domain organization of L. crispatus adhesion and colonization factors. A representative member of each OrthoMCL-group is presented graphically. The larger colored blocks represent adhesion or colonization related PFAM-domains and the thinner blocks other domains. The names of each color-coded domain are given at the bottom of the picture. (PDF 145 KB) [file 12864_2014_6771_MOESM10_ESM.pdf]
